# Reach and efficacy of the eHealth application Oncokompas, facilitating partners of incurably ill cancer patients to self-manage their caregiver needs: a randomized controlled trial

**DOI:** 10.1007/s00520-022-07441-4

**Published:** 2022-11-11

**Authors:** Anouk S. Schuit, Michelle M. Rienks, Nienke Hooghiemstra, Femke Jansen, Birgit I. Lissenberg-Witte, Pim Cuijpers, Irma M. Verdonck-de Leeuw, Karen Holtmaat

**Affiliations:** 1 Department Clinical, Neuro and Developmental Psychology, Vrije Universiteit Amsterdam, Van Der Boechorststraat 7-9, Amsterdam, The Netherlands; 2 Cancer Center Amsterdam, Treatment and Quality of Life, Amsterdam, The Netherlands; 3 Amsterdam Public Health, Mental Health, Amsterdam, The Netherlands; 4 Department of Otolaryngology-Head and Neck Surgery, Amsterdam UMC Location Vrije Universiteit Amsterdam, De Boelelaan 1117, Amsterdam, the Netherlands; 5 Department of Epidemiology and Data Science, Amsterdam UMC Location Vrije Universiteit Amsterdam, De Boelelaan 1117, Amsterdam, The Netherlands

**Keywords:** eHealth, Palliative care, Caregiving, Partners, Incurable cancer, Caregiver burden

## Abstract

**Purpose:**

Many partners of incurably ill cancer patients experience caregiver burden. The eHealth application “Oncokompas” supports these partners to manage their caregiver needs and to find optimal supportive care for themselves. The aim of this randomized controlled trial (RCT) was to investigate the reach of Oncokompas and its efficacy on caregiver burden, self-efficacy, and health-related quality of life (HRQOL).

**Methods:**

The reach was estimated based on eligibility, participation rate, and an evaluation of the recruitment process. Efficacy on caregiver burden was measured using the Caregiver Strain Index + (CSI +). Secondary outcomes were self-efficacy (General Self-Efficacy Scale (GSE)) and HRQOL (EQ-5D VAS). Assessments were scheduled at baseline, 2 weeks after randomization and 3 months after baseline. Linear mixed models were used to compare longitudinal changes between the experimental and control group from baseline to the 3-month follow-up.

**Results:**

The reach, in terms of eligibility and participation rate, was estimated at 83–91%. Partners were most likely reached via palliative care consultants, patient organizations, and palliative care networks. In the one-and-a-half-year recruitment period and via the 101 organizations involved, 58 partners were included. There were no significant effects of Oncokompas on caregiver burden, self-efficacy, or HRQOL.

**Conclusion:**

The reach of Oncokompas among interested individuals was high, but the difficulties that were encountered to include partners suggest that the reach in real life may be lower. This study showed no effect of Oncokompas on caregiver burden, self-efficacy, or HRQOL in partners of incurably ill cancer patients.

**Relevance:**

The results of this study may be used in the process of developing, efficacy testing, and implementing eHealth applications for caregivers of incurably ill cancer patients.

**Trial registration:**

Netherlands Trial Register identifier: NTR7636/NL7411. Registered on November 23, 2018 (https://www.trialregister.nl/).

## Introduction

There is convincing evidence that informal caregiving for an incurably ill cancer patient is associated with physical, psychological, and social problems and that these problems negatively impact aspects of health-related quality of life (HRQOL) of informal caregivers [1–14]. Caregiver burden can be defined as “a multidimensional biopsychosocial reaction resulting from an imbalance of care demands relative to caregivers’ personal time, social roles, physical and emotional states, financial resources, and formal care resources given the other multiple roles they fulfill” [15]. There is a growing interest in healthcare resources to support informal caregivers of incurably ill patients. Many informal caregivers do not use these healthcare options. For instance, because they are unaware or unconcerned that their own HRQOL is being compromised, they are unaware of the available healthcare resources, they do not have access to these resources at the moment they need them, or they may feel that focusing at their own needs is at the expense of the patients’ needs [16–21]. Delivering interventions through the Internet may help to reach a greater number of informal caregivers [17, 22–24].

The eHealth self-management application Oncokompas was developed to support partners of incurably ill cancer patients to adopt an active role in improving their own HRQOL and to find optimal supportive care if needed. Oncokompas specifically targets partners of incurably ill cancer patients to optimally tailor information, advice, and supportive care options to their situation. Oncokompas helps partners of incurably ill cancer patients to monitor their own HRQOL using patient-reported outcome measures (PROMs), followed by automatically generated tailored feedback, self-care advice, and advice on supportive care services. The application is tailored to the partner’s personal characteristics and preferences and can be accessed 24/7 [25].

The aim of this randomized controlled trial (RCT) was to investigate the reach and efficacy of Oncokompas as a digital self-management instrument on caregiver burden, self-efficacy, and HRQOL among partners of patients with incurable cancer. The reach and efficacy were evaluated in the context of the RE-AIM model [26]. The reach was estimated based on eligibility, participation rate, and an evaluation of the recruitment process of this RCT. It is expected that Oncokompas reaches about 45% of the partners and that using Oncokompas helps partners to reduce caregiver burden and to increase self-efficacy and HRQOL [27, 28].

## Methods

### Study design

A prospective RCT with two parallel groups was conducted to investigate the reach and efficacy of Oncokompas among partners of patients with incurable cancer. Partners in the intervention group got access to Oncokompas directly after completing the baseline questionnaire, and partners in the control group got access after three months (i.e., after completing the last questionnaire). Outcome measures were collected at baseline (t0), 2 weeks after randomization (t1), and 3 months after the baseline measurement (t2).

The study protocol was approved by the Medical Ethics Committee of VU University Medical Center (2018.517). This trial was registered in the Netherlands Trial Register (NTR7636/NL7411), and the study protocol was published previously [25]. All participants provided written informed consent. The CONSORT guidelines (CONsolidated Standards of Reporting Trials) were used to report on this trial [29].

### Study population

Inclusion criteria were as follows: being an adult (aged ≥ 18 years) partner of an incurably ill cancer patient and having access to an e-mail address. Partners self-identified as partner. There were no restrictions with regard to their marital status, living situation, duration, or quality of their relationship. Patients were defined as incurably ill if their partner reported that the patient did not have curative treatment options. Partners were excluded when they had severe cognitive impairments or when they had a poor understanding of the Dutch language. They were also excluded when their partner with cancer already used Oncokompas for patients with incurable cancer.

### Recruitment

A multi-component recruitment strategy was followed in which healthcare professionals in various settings were asked to place and spread recruitment materials and to inform partners of incurably ill cancer patients on the study (Table 1). Partners could also contact the researchers directly by using the reply form at the Oncokompas website or by e-mailing the researchers. Recruitment materials consisted of leaflets in waiting rooms and offices of healthcare providers, and online advertising on websites, newsletters, and social media. The contact details of the researcher and URL of the Oncokompas website (www.oncokompas.nl) were mentioned in all materials.

**Table 1:** Overview of parties involved in recruiting participants

Type of organization	Approached	Agreed to participate		Declined		No response		Main reasons to decline
	*N*	*n*	%	*n*	%	*n*	%	
General practitioner	288	11	4	13	5	264	92	No time and interest
Hospital	28	3	11	8	29	17	61	No time or already involved in other studies
Home care organization	42	1	2	6	14	35	83	Not in contact with (many) partners of incurably ill cancer patients
Center for supportive cancer care	68	26	38	0	0	42	62	
Patient organization	23	11	48	7	30	5	22	Not in contact with partners of incurably ill cancer patients
Informal care organization	51	7	14	10	20	34	67	Already involved in other studies
Informal care consultant	86	16	19	18	21	52	60	Not in contact with partners of incurably ill cancer patients
Palliative care network	41	17	41	3	7	21	51	Already involved in other studies
Palliative care consultant	5	5	100	0	0	0	0	
Elderly association	24	4	17	2	8	18	75	
Total	656	101	15	67	10	488	74	

### Study procedures

Individuals who expressed interest in participating in this study were contacted by a researcher to be further informed about the study. Eligible partners received an information letter and informed consent form. After signing informed consent, partners received the baseline questionnaire by e-mail. After completion of the baseline questionnaire, partners were randomly assigned to a study arm. Partners randomized to the intervention group received an invitation e-mail for Oncokompas to activate their personal account. Partners randomized to the control group received this e-mail after completion of the third questionnaire (t2).

### Randomization

Partners were randomized in a 1:1 ratio. Block randomization was used with a random block length of four, six, or eight. Stratification was not applied. The randomization scheme was computer-generated, created by a researcher not involved in the study, who also performed the allocation of participants. Neither the researcher, and, because of the nature of the intervention, participants could not be blinded.

### Wait list control group

All partners received care as usual during their participation in the wait list control group. Care as usual was defined as all care provided by healthcare professionals regardless of study participation. Care as usual may for example consist of consults with a medical specialist, general practitioner, psychotherapist, or physiotherapist.

### Intervention

**Fig. 1:**
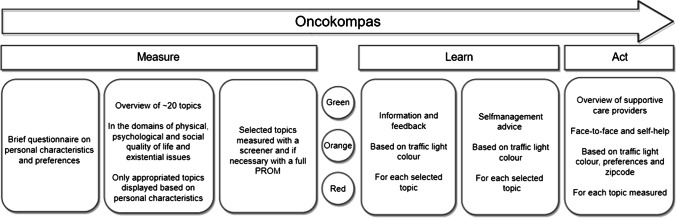
Overview of the eHealth application Oncokompas

Oncokompas is an eHealth self-management application, consisting of three steps: measure, learn, and act (Fig. 1). Previously, a version of Oncokompas has been developed for cancer patients during or after curative treatment [27] and for incurably ill cancer patients [28]. All versions were developed using a stepwise, iterative, and participatory approach, actively involving end users and health care professionals [30]. The development process consisted of six steps: (1) selection of topics, (2) selection of PROMs, (3) determining cut-off scores, (4) drafting information texts, (5) drafting self-management advice texts, and (6) selecting health care options. This means that within the existing framework of Oncokompas, new content for partners of incurably ill patients was developed. Each step was carried out by researchers (AS, NH, and KH) and discussed with an expert team consisting of health care professionals, partners of incurably ill cancer patients, and researchers. Previously, two RCTs were conducted on the efficacy of Oncokompas among cancer survivors [27] and incurably ill cancer patients [28] that did not show an effect at patient activation (primary outcome), but did show effects on HRQOL and specific symptoms among survivors.

In the first step of Oncokompas, “measure,” partners complete a questionnaire on their personal characteristics, used to automatically display the topics appropriate for this individual (e.g., when someone is retired, the topic about “work” will not be shown). Then, partners can select which topics they want to address within Oncokompas (e.g., fatigue, loneliness, or financial problems). The topics target four domains of quality of life: physical, psychological and social functioning, and existential issues. Subsequently, PROMs are used to measure partners’ functioning on the selected topics [25]. In the step, “learn”, Oncokompas provides information and feedback on partners’ outcomes, tailored to their personal characteristics and preferences. Using a traffic-light system (green, orange, and red), partners get an overview of their well-being per topic. A green score means that the partner is doing well on this topic, an orange score means that this topic *could* use attention and support, and a red score means that this topic *needs* attention and support. Then, Oncokompas provides comprehensive self-care advice. Lastly, within the step “act,” partners receive a personal overview of supportive care options for themselves, with options for professional guidance when needed.

### Study measures

Caregiver burden was assessed using the Caregiver Strain Index + (CSI +). The CSI + measures the self-reported burden that informal caregivers experience as a result of caring for their loved ones (13 items), as well as positive and rewarding experiences as a result of informal caregiving (5 items). Response options are “yes” (coded as 1 for the negative items and − 1 for the positive items) and “no” (always coded as 0). The total score range is − 5 to 13. A higher score indicates more caregiver burden. The CSI + does not have a cut-off score. The total CSI + score was analyzed, as well as the negative and positive items separately. In the separate analyses, a higher score indicates more caregiver burden. In contrary to the way positive items of the CSI + were coded (as − 1), in the separate analyses, positive items were coded as 1, so that a higher score indicates a more positive caregiver experience [31,32).

Self-efficacy was assessed using the General Self-Efficacy Scale (GSE). The GSE is a 10-item self-report questionnaire, assessing how a person deals with difficult situations in life. The items have a 4-point Likert scale, ranging from 1 (not at all true) up to 4 (exactly true). The total score is calculated by adding up the scores on the 10 items and ranges from 10 to 40. A higher score indicates a greater sense of self-efficacy. The GSE does not have a cut-off score. The international average GSE score in the general population is 29.55 [33, 34].

HRQOL was measured using the visual analogue scale (VAS) of the self-report questionnaire EuroQol-5D (EQ-5D). The VAS ranges from 0 to 100, in which 100 indicates the best imaginable health state. The norm score of general Dutch citizens from 55 to 64 years is 80.7 [35, 36].

Partners’ and patients’ sociodemographic and health-related characteristics were assessed at baseline using a study specific questionnaire.

### Sample size

To demonstrate an increase on the CSI + of at least 0.5 standard deviations in the intervention group compared to the control group (i.e., between group change of 0.5 SD) between t0 and t2 as statistically significant in a one-tailed test using a significance level of 5% (alpha = 0.05) and a power of 80% (1 − *β* = 0.80), 51 participants were required at t2 in each study arm. Anticipating a drop-out rate of 25% between t0 and t2, 68 participants per study arm needed to be included at t0. In total, a sample of 136 participants was needed.

### Statistical analyses

Reach was estimated based on eligibility and participation rate. Eligibility rate was calculated as the number of eligible partners divided by the number of partners who were informed on the study after they expressed interest in Oncokompas. The participation rate was calculated by dividing the number of included partners by the number of eligible partners.

Descriptive statistics were used to describe the recruitment process, sociodemographic and health-related characteristics of the partner and the patient, and the outcome measures at baseline.

Linear mixed models (LMM) were used to compare longitudinal changes in primary and secondary outcome measures in both study arms between t0, t1, and t2. Fixed effects were used for study arm, measurement, and their two-way interaction, and a random intercept for subjects. All analyses were conducted according to the intention-to-treat principle. Missing data was not imputed as LMM accounts for missing data. As sensitivity analyses, the LMM were repeated comparing participants who used Oncokompas as intended with participants in the control condition. Usage as intended was defined as completing the steps “measure” and “learn” at least for one topic.

All analyses were performed using the IBM Statistical Package for the Social Sciences (SPSS) version 27 (IBM Corp., Armonk, NY USA). A *p*-value of < 0.05 was considered significant for all analyses.

## Results

### Reach: recruitment process

During the recruitment process, 656 organizations involved in palliative care were asked to participate in recruiting partners (Table 1). In total, 101 agreed to participate (15%), 67 declined (10%), and the majority did not respond (*n* = 488, 74%). Main reasons for organizations that declined were the following: having no time, already being involved in other studies, or not having partners of incurably ill cancer patients in their care. The types of organization that most often agreed to participate, in terms of percentages, were palliative care consultants (100%), patient organizations (48%), palliative care networks (41%), and centers for supportive cancer care (38%). The types of organizations that were least likely to participate were home care organizations (2%), general practitioners (4%), and hospitals (11%). Despite all efforts, after recruiting for almost one and a half year, the inclusion of partners lagged behind considerably. Part of the study (March-August 2020) took place during the COVID-19 pandemic. Due to the national lockdown in the Netherlands, many organizations were not able to continue their services as they were used to. Reaching the target of 136 included partners was no longer considered feasible, and therefore, the study stopped in September 2020. The 101 parties involved in the recruitment process led to 93 individuals expressing interest in Oncokompas and 58 inclusions.

### Reach: eligibility and participation rate

**Fig. 2:**
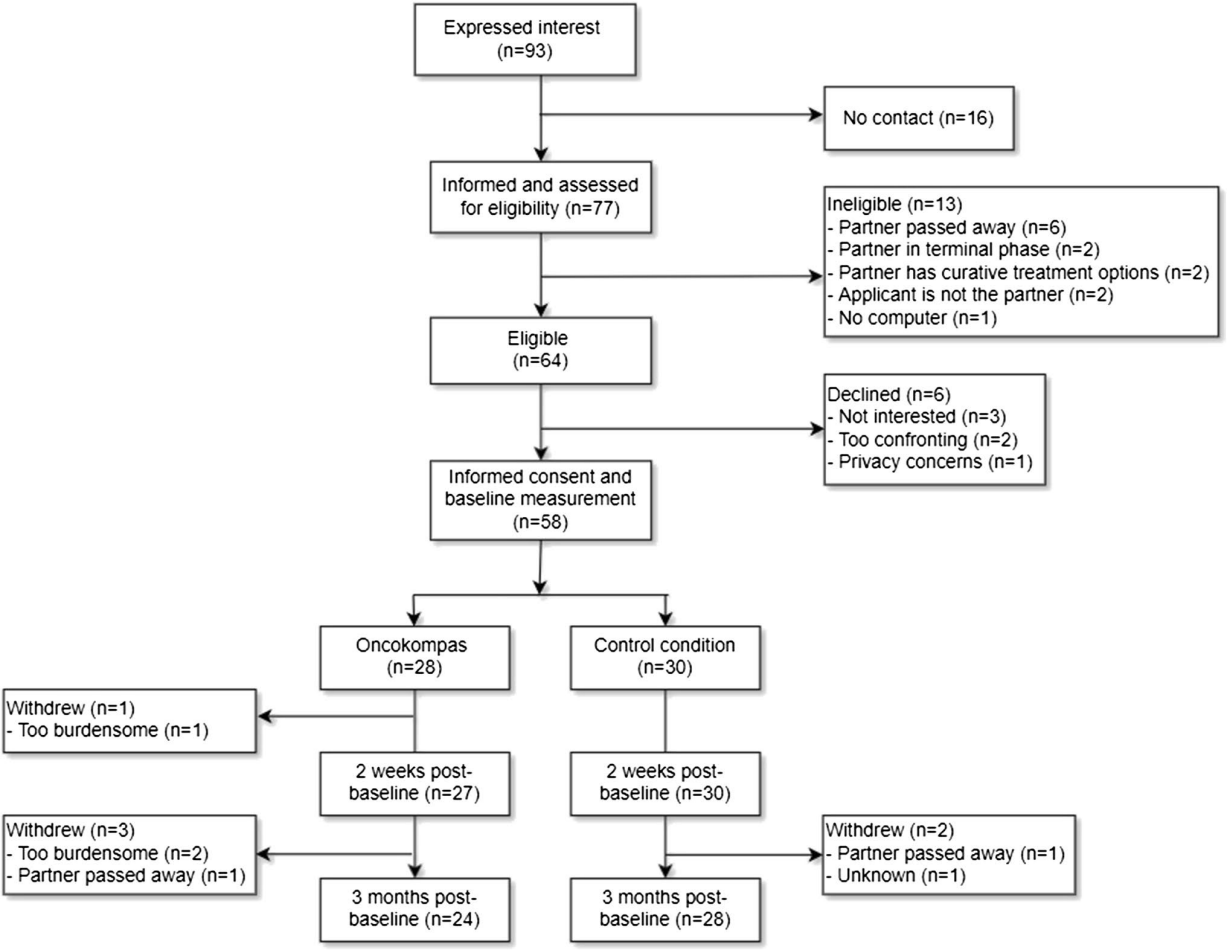
Flow diagram

Between March 2019 and August 2020, 93 individuals expressed interest in Oncokompas. Sixteen of them could not be contacted. The other 77 were informed about the study, of which 64 were eligible (eligibility rate 83%). Reasons for ineligibility were as follows: patient (the partner’s partner) passed away (*n* = 6), patient was in the terminal phase of the disease (*n* = 2), patient still had options for curative treatment (*n* = 2), applicant was not the partner but another informal caregiver (*n* = 2), and having no computer (*n* = 1). Of the 64 eligible partners, 58 agreed to participate in the study (participation rate 91%). Reasons for not agreeing to participate were the following: not being interested (*n* = 3), participation being too confronting (*n* = 2), and having privacy concerns (*n* = 1). The flow diagram of the study is shown in Fig. 2.

### Efficacy

Of the 58 included partners, 28 were allocated to the intervention group and 30 to the control group (Fig. 1). Mean age was 57 years, and two-thirds (67%) were female. The majority had children (86%), were highly educated (55%), and employed (60%). Almost half of the partners (45%) reported no comorbidities (Table 2). The patients that the partners were caring for were on average 59 years of age and their health (as perceived by the partner) was on average 4.7 on a scale of 0 to 10. Most of the patients had lung cancer (19%) or a brain tumor (17%), and received treatment primarily directed at the disease (as opposed to treatment primarily directed at reducing symptoms) (72%). Fifty-one percent were diagnosed with cancer more than 2 years ago (Table 3).

**Table 2:** Sociodemographic characteristics of the study participants

	Total group (*n* = 58)		Control group (*n* = 30)		Intervention group (*n* = 28)	
	*n*	%	*n*	%	*n*	%
Age in years						
Mean (SD)	57 (11)	-	58 (13)	-	57 (10)	-
Gender						
Male	19	33	10	33	9	32
Female	39	67	20	67	19	68
Education level						
Low	7	12	2	7	5	18
Medium	19	33	11	37	8	29
High	32	55	17	57	15	54
Children						
Yes	50	86	27	90	23	82
No	8	14	3	10	5	18
Employed						
Yes	35	60	17	57	18	64
No	23	40	13	43	10	36
Comorbidities						
None	26	45	13	43	13	46
One comorbidity	18	31	11	37	7	25
Multiple comorbidities	14	24	6	20	8	29

**Table 3:** Sociodemographic and clinical characteristics of the patients

	Total group (*n* = 58)		Control group (*n* = 30)		Intervention group (*n* = 28)	
	*n*	%	*n*	%	*n*	%
Age in years						
Mean (SD)	59 (12)	-	61 (14)	-	57 (10)	-
Gender						
Male	40	69	21	70	19	68
Female	18	31	9	30	9	32
Health as perceived by partner (0–10)	4.7 (1.9)		4.9 (1.9)		4.5 (2.0)	
Tumor site						
Lung	11	19	5	17	6	21
Brain	10	17	5	17	5	18
Prostate	6	10	4	13	2	7
Colon	6	10	2	7	4	15
Breast	5	9	3	10	2	7
Hematological	4	7	1	3	3	11
Other	14	24	9	30	5	18
Multiple	2	3	1	3	1	4
Time since diagnosis						
< 1 month	4	7	3	10	1	4
< 6 months	7	12	3	10	4	14
< 2 years	17	29	8	27	9	32
> 2 years	30	51	16	53	14	50
Treatment target						
Cancer	42	72	22	73	20	71
Symptoms	7	12	5	17	2	7
No treatment	9	16	3	10	6	21

Results of the linear mixed model analyses are shown in Table 4. No significant difference was found in the course of caregiver burden (CSI +) in the intervention group, compared to the control group. The estimated difference in change from t0 to t2 was 0.3 points (90% CI − 0.8–1.5). This means that the estimated change in the intervention group (t0–t2) was 0.3 points higher than in the control group. The *p*-value of the interaction between the study arm and the time of assessment was 0.64.

**Table 4:** Mean scores per group per assessment and results of the linear mixed model analyses on the primary and secondary outcome measures

	Baseline (t0)		2 weeks follow-up (t1)		3 months follow-up (t2)		Estimated difference in change between T0 and T2 (90% CI)	*P*-value two-way interaction
	*n*	Mean (SD)	*n*	Mean (SD)	*n*	Mean (SD)		
Caregiver Strain Index (CSI +)								0.64
Intervention	28	2.8 (3.4)	25	2.5 (3.2)	22	2.5 (2.6)	0.3 (− 0.8–1.5)	
Control	30	4.2 (3.2)	30	4.6 (3.0)	24	4.3 (3.0)		
Caregiver Strain Index (CSI + negative items)								0.53
Intervention	28	7.1 (3.0)	25	6.8 (2.7)	22	7.0 (2.4)	0.2 (− 0.7–1.1)	
Control	30	8.3 (2.7)	30	8.5 (2.5)	24	8.3 (2.4)		
Caregiver Strain Index (CSI + positive items)								0.79
Intervention	28	4.4 (0.8)	25	4.2 (0.7)	22	4.5 (0.6)	− 0.2 (− 0.6–0.2)	
Control	30	4.0 (0.9)	30	3.9 (1.0)	24	4.0 (1.0)		
General self-efficacy (GSE)								0.77
Intervention	28	30.8 (6.2)	26	30.8 (6.0)	24	30.7 (5.7)	− 0.1 (− 1.6–1.3)	
Control	30	31.2 (3.5)	30	31.7 (4.1)	28	31.0 (3.3)		
HRQOL* (EQ-5D VAS)								0.24
Intervention	28	72.8 (16.7)	26	77.0 (13.2)	24	74.0 (19.2)	1.0 (− 6.2–8.2)	
Control	30	72.8 (14.3)	30	71.2 (15.0)	24	75.0 (12.1)		

*HRQOL: health-related quality of life.

Also, the course of caregiver burden (negative items of the CSI +), positive caregiving experience (positive items of the CSI +), self-efficacy (GSE), and HRQOL (EQ-5D VAS) did not differ significantly between partners randomized into the intervention or wait list control group.

### Usage of Oncokompas

Of the 28 partners in the intervention group, 27 activated their account and 22 of them (81%) used Oncokompas as intended during the three-month follow-up period. The median number of logins among intended users was 3 (interquartile range (IQR) 2–4). The course of all outcome measures was not significantly different between partners who used Oncokompas as intended and partners in the control condition.

## Discussion

This study examined the reach and efficacy of the eHealth self-management application Oncokompas, among partners of patients with incurable cancer. The reach was estimated at 83–91%. There was no significant effect on caregiver burden, self-efficacy, or HRQOL of the partners.

In this study, reach was defined as a combination of the eligibility and participation rate of individuals with an expressed interest in Oncokompas, complemented by an evaluation of the recruitment process. While the eligibility and participation rate were high, the difficulties that were encountered to include partners suggest that in real life the reach may be lower. In previous studies, the reach of Oncokompas was estimated to be 45–68% in cancer survivors [27] and 63% in patients with incurable cancer [28]. Main reasons for not reaching cancer survivors were the following: wanting to leave the period of being ill behind, no symptom burden, or lacking computer skills [37]. Main reasons for not reaching patients with incurable cancer were as follows: participation being too confronting or lacking computer skills [28]. In the present study, various online channels were used to recruit partners, which may explain that a lack of computer skills was not a main reason for not reaching the target population, and which may have overestimated the eligibility and participation rate. In contrast to the studies among cancer survivors and patients, where recruitment took place in hospitals solely, partners were most likely reached via palliative care consultants, palliative care networks, and patient organizations and less via hospitals. A hospital-based recruitment strategy might have been more successful, but was not feasible for the present study, because in parallel incurably ill cancer patients were recruited in the hospital for the study on the efficacy of Oncokompas for patients [28]. Unfortunately, during the present study, all recruitment channels were affected by the national lock-down due to the COVID-19 pandemic.

Based on the results of this study, it cannot be concluded that Oncokompas decreases caregiver burden, and increases self-efficacy or HRQOL in partners of incurably ill cancer patients. A meta-analysis suggests that caregiver burden is hardly affected by eHealth interventions [38]. The absence of significant effects of Oncokompas is in line with a recent study on the efficacy of the version of Oncokompas for incurably ill cancer patients [28], but not with a large study on the efficacy of the version of Oncokompas for cancer survivors [27]. Oncokompas for cancer survivors differs from the other two versions of Oncokompas, in that it has several tumor-specific modules and its effects were mainly found among tumor-specific symptoms. Oncokompas for partners is tailored to the characteristics and preferences of the partner, but may need to be further tailored to the specific demands of caring for a patient with a specific tumor type [39]. At the same time, Oncokompas may have had specific effects for partners, such as feeling less fatigued or lonely, but these specific effects may not have been captured by the generic outcome measures used in this study. The absence of significant effects of Oncokompas may also be explained by the low number of logins among the 81% who used Oncokompas as intended (median number of logins = 3) or by the finding in a previous study that about 60% of the Oncokompas users feel that the tailored content is still not applicable to their situation [37].

There are also some limitations that may explain these results. First, the sample size was smaller than intended, and therefore, the power to detect changes in the outcome measures was insufficient. Second, Oncokompas was developed before the COVID-19 pandemic, but the RCT was largely conducted during the pandemic. There might have been an effect on the personalized supportive care options as provided in the third step within Oncokompas (the “act” component). When a user has a red score on a topic, the feedback always included the advice to contact a healthcare professional. These contacts may have changed from face-to-face contact to contact through telehealth during the COVID-19 pandemic. Third, the follow-up period of three months might have been too short to measure effects that take more than three months (e.g., psychotherapy or physiotherapy). A fourth explanation could be that 51% of the participants were caring for a patient diagnosed more than two years ago. These partners may have already learned to cope with the situation and did not need Oncokompas at that moment anymore. In any case, it may be better to provide access to Oncokompas at an early stage, for instance, shortly after a diagnosis of incurable cancer. Such an approach would also fit well into advanced cancer care planning [40].

Alongside this RCT, a cost-utility analysis was planned. It was expected that Oncokompas would improve quality adjusted life years at acceptable costs, compared to the wait list control group [25]. Because the sample size was smaller than expected, this cost-utility analysis was deemed not to be feasible and was not carried out. Nonetheless, this project generated new knowledge on the reach and efficacy of an eHealth self-management intervention among partners of incurably ill cancer patients.

In conclusion, the reach of Oncokompas among interested individuals was high, but the difficulties that were encountered to include partners suggest that the reach in real life may be lower. This study showed no effect of Oncokompas on caregiver burden, self-efficacy, or HRQOL in partners of incurably ill cancer patients.

IMVdL, KH, and AS developed the eHealth self-management application Oncokompas and were involved in the design of the study. AS, KH, and IMVdL coordinated the study and were responsible for all aspects of local organization which they discussed in monthly meetings. IMVdL was the leading researcher responsible for the trial, overseeing conduct and progress. AS and NH were responsible for day-to-day support of the trial and aware of the outcome of the allocation during randomization. MR, KH, and BLW performed the data analyses. AS, MR, KH, and IMVdL drafted the manuscript. NH, FJ, BLW, and PC critically revised the manuscript. All authors read and approved the final manuscript.

## Data Availability

The datasets analyzed during the current study will be available in the EASY repository of DANS-KNAW after completion of the thesis that will be written reserving the generated data. The (intellectual) property rights with regard to the generated data will reside at the Vrije Universiteit Amsterdam. Interested parties can request a non-exclusive license for research and educational purposes. The data will be available up to 15 years after the end of the study.

## Abbreviations

*CSI*: Caregiver Strain Index
*EQ-5D*: EuroQol 5-Dimensions questionnaire
*GSE*: General Self-Efficacy
*HRQOL*: Health-related quality of life
*LMM*: Linear mixed models
*PROMs*: Patient-reported outcome measures
*RCT*: Randomized controlled trial
*CONSORT*: CONsolidated Standards of Reporting Trials
